# Bacterial and Fungal Community Structures in Loess Plateau Grasslands with Different Grazing Intensities

**DOI:** 10.3389/fmicb.2017.00606

**Published:** 2017-04-07

**Authors:** Huhe, Xianjiang Chen, Fujiang Hou, Yanpei Wu, Yunxiang Cheng

**Affiliations:** ^1^Soil Fertilizer and Water-Saving Institute, Gansu Academy of Agricultural SciencesLanzhou, China; ^2^The Ministry of Agriculture in Gansu Province Cultivated Land Conservation and Agricultural Environmental Science Observation Experiment StationsWuwei, China; ^3^State Key Laboratory of Grassland Agro-ecosystems, College of Pastoral Agriculture Science and Technology, Lanzhou UniversityLanzhou, China

**Keywords:** Loess Plateau grassland, grazing intensity, bacterial community, fungal community, Illumina MiSeq platform

## Abstract

The Loess Plateau of China is one of the most fragile ecosystems worldwide; thus, human production activities need to be conducted very cautiously. In this study, MiSeq high-throughput sequencing was applied to assess the relationship between bacterial and fungal community structures and changes in vegetation and soil physical and chemical properties induced by grazing, in four grasslands with different levels of grazing intensity (0, 2.67, 5.33, and 8.67 sheep/ha) in the semiarid region of the Loess Plateau. The relative abundances of the bacterial community in the grasslands with 2.67 and 5.33 sheep/ha were significantly higher than those in grasslands with 0 and 8.67 sheep/ha, and the fungal diversity was significantly lower for grasslands with 2.67 sheep/ha than for the other grasslands. Redundancy analysis (RDA) showed that plant biomass, nitrate, and total nitrogen have significant effects on bacterial community structure, whereas nitrate and total nitrogen also significantly affect fungal community structure. Variation partitioning showed that soil and plant characteristics influence the bacterial and fungal community structures; these characteristics explained 51.9 and 52.9% of the variation, respectively. Thus, bacterial and fungal community structures are very sensitive to grazing activity and change to different extents with different grazing intensities. Based on our findings, a grazing intensity of about 2.67 sheep/ha is considered the most appropriate in semiarid grassland of the Loess Plateau.

## Introduction

The Loess Plateau of China is located in the upper middle stretches of the Yellow River and includes the 624,000 km^2^ loess deposition area west of the Taihang Mountains, east of the Riyue Mountain, north of the Qin Mountains, and south of the Yin Mountains. The plateau is the most severely eroded area in the world; over 60% of the land is affected by erosion due to natural (topography, climate, soil, and vegetation) as well as anthropogenic (unreasonable production and construction) causes, and the average annual soil loss is about 2000–2500 t/km^2^ (Shi and Shao, 2000). Soil erosion, which is mainly caused by grassland degradation, has severely damaged the land resources and the ecosystem of the Loess Plateau.

Grassland soil degradation is a reverse evolutionary ecosystem process instigated by human activities as well as natural factors. One of the most important causes is overgrazing (Lusigi and Glaser, 1984; Sinclair and Fryxell, 1985; Hammond, 1992). Livestock behaviors such as feeding, trampling, and fouling lead to changes in the plant community and the physical and chemical properties of soil. Generally, the pasture recovery ability and total biomass decrease with increasing stocking rate (Christiansen and Srejcor, 1988). However, some studies have suggested that moderate grazing can actually improve grassland, yielding higher plant diversity and productivity (Mcintyre et al., 2003; Ruifrok et al., 2014). In addition, grazing compacts the soil (Weigel et al., 1990), reduces the soil water-holding capacity and water potential (Kobayashi et al., 1997), and affects soil humus and nitrogen accumulation (Severson and Debano, 1991).

Soil microbes are an important component of grassland ecosystems; they regulate matter cycling and promote energy flow in the ecosystem. Owing to their wide biochemical activities, they act as stores and sources of active soil nutrients (Smith and Paul, 1990). As a result, microbes are very sensitive to environmental changes (Nielsen and Winding, 2002), and they adapt to different environments. Grazing activities affect the soil microbial community structure primarily by changing the vegetation and soil physical and chemical properties. Moreover, interactions exist between plants and microbes; plants produce a large variety of root exudates and secretions, including organic substances, enzymes, and polysaccharides, which can benefit or inhibit microbes. In addition, plants can distinguish compounds from various microbial origins and adjust their defense and growth responses on the basis thereof. Conversely, microbes can initiate their colonization strategies in the rhizosphere and synthesize canonical plant growth regulators such as auxins and cytokinins when appropriate host plants are detected (Ortiz-Castro et al., 2009).

For the purpose of clear and reasonable grazing management, an increasing number of studies have focused on the effects of grazing on soil microbial communities. Some studies have found that soil microbial biomass and diversity increase along a gradient of increasing grazing intensity (Ruess and McNaughton, 1987; Bardgett et al., 1997; Qi et al., 2011; Gou et al., 2015); Jangid et al. (2008) and Zhou et al. (2010) suggested that soil microbe diversity is maximal at an intermediate grazing intensity. In addition, Patra et al. (2005) found no obvious differences in fungal and bacterial abundances between light grazing and intensive grazing in seminatural grassland. In studies of salt marsh and sand-dune grassland, Ford et al. (2013) discovered that grazing behavior has a limited impact on soil microbial community structure, which was instead primarily affected by the type of grassland. Together, these studies indicate that results tend to vary between regions with different environments. The relationship between biodiversity and disturbance is affected by geographic location (Death and Barquín, 2012), and different soil types (Arenic Endoaqualfs and Arenic Glossaqualfs) are exposed to different grazing pressures, which cause significant differences in bulk density, organic matter, and carbon mineralization rate in Arenic Endoaqualfs as compared to that in Arenic Glossaqualfs (Wang et al., 2006). For the easily eroded grassland regions of the Loess Plateau, which are highly susceptible to the effects of wind and water, the development and enforcement of reasonable grazing management systems are even more important. A 16S rRNA gene clone library was previously generated to analyze the bacterial community structure of soils with different grazing intensities in the plateau, and it was found that the biomass and diversity increased along a gradient of increasing grazing intensity (Gou et al., 2015). However, this library method detects only a very limited group of microbes, which is a serious limitation with respect to gaining an overall scientific understanding of the soil microbial diversity (McCaig et al., 2001; Nacke et al., 2011).

This study adopted high-throughput sequencing methods to sequence partial bacterial 16S rRNA and fungal 18S rRNA genes to compare the bacterial and fungal community structures in four types of Loess Plateau semiarid grasslands with different grazing intensities and to elucidate their interactions with vegetation and soil physical and chemical properties. A better understanding of the biotic and abiotic components of grasslands with different grazing intensities provides a theoretical basis for the management of Loess Plateau grazed grassland.

## Materials and Methods

### Study Site and Sampling

The study site was a grassland located in the Loess Plateau (37.12° N, 106.82° E, 1650 m elevation), Huanxian County, Gansu Province, China, with a typical continental monsoon climate. The location has a mean annual temperature of 7.1°C, mean annual precipitation of 359.3 mm, mean annual evaporation of 1993.3 mm, and approximately 70% of annual rainfall occurring from June to September. The study site was divided into four zones with different grazing intensities (0, 2.67, 5.33, and 8.67 sheep/ha; designated S0, S2.67, S5.33, and S8.67). Each grazing regimen was randomly arranged into three repeats; each experimental plot was 50 m × 100 m in size. Rotational grazing was permitted in a small experimental area from mid-June to mid-September of every year, with 10 days of grazing per rotation and three grazing rotations in total. Tan sheep had been grazed continuously for 13 years at the time sample collection was started. In August 2014, soil samples were collected and plant community characteristics were investigated. In each sampling area, 3 quadrats (1 m × 1 m) were randomly selected, the plant species in each quadrat were identified, and the cover was measured. Aboveground clippings of plants were collected, dried at 60°C for 48 h, and weighed. Next, five surface soil samples (0–10 cm depth) were randomly collected from each quadrat and immediately mixed into a single soil sample. Visible matter, such as loose gravel and plant debris, were removed and divided into two parts. One part was passed through a 2-mm sieve into a 15-mL centrifuge tube, placed into a liquid nitrogen canister, transferred to the laboratory, and stored in a -80°C freezer for microbial analyses. The other part was sealed in a plastic zip-top bag and transferred to the laboratory in an icebox to be used for the determination of soil physical and chemical properties.

### Determination of Physical and Chemical Properties of the Soil

All of the nine replicate soil samples collected from each grazing zone were analyzed for physical and chemical properties. Soil pH was determined using a 1:2.5 soil/water mixture. After the mass was stabilized by drying at 105°C for 24 h, the water content of the soil was calculated. Water and inorganic carbon were removed by reacting the samples with phosphoric acid (Schumacher, 2002), after which soil organic carbon and total nitrogen were measured using the NC analyzer dry combustion method (Sumigraph NC-900; Sumika Chemical Analysis Service, Tokyo, Japan). For chemical analyses, soil was digested in H_2_SO_4_ for total nitrogen and in H_2_SO_4_/HCl for total phosphorus, extracted with KCl for nitrate and ammonium, and analyzed using an automated flow analysis instrument (FIAstar 5000 Analyzer; Foss Tecator, Hillerød, Denmark).

### DNA Collection and High-Throughput Sequencing

Genomic DNA was isolated from 0.5 g of each pooled soil sample from each sample plot (*n* = 36) with the PowerSoil DNA Isolation Kit (Mo Bio Laboratories, Solana Beach, CA, USA) per the manufacturer’s instructions. The extracts of three technical repeats were mixed into a single DNA sample. Extracted genomic DNA was detected by 1% agarose gel electrophoresis. PCR was carried out on a GeneAmp 9700 PCR system (Applied Biosystems, Foster City, CA, USA). Based previous reports, the primers 338F-806R (Huws et al., 2007) and 817F-1196R (Rousk et al., 2010) were used for the 16S rRNA and 18S rRNA genes, respectively. Amplified products were detected by 2% agarose gel electrophoresis and recovered from the gel, using the AxyPrep DNA gel extraction kit (Axygen Biosciences, Union City, CA, USA), washed with Tris-HCl, and verified by 2% agarose gel electrophoresis. PCR products were quantified using the QuantiFluor^TM^-ST Fluorometer (Promega Biotech, Beijing, China), and the samples were adjusted as needed for sequencing. Sequencing was conducted by Shanghai Majorbio Bio-pharm Technology (Shanghai, China), using an Illumina MiSeq platform (San Diego, CA, USA).

### Sequence Analysis

Obtained DNA sequences were processed using the mothur software (Schloss et al., 2009). Sequences shorter than 200 bp, ambiguous bases, and sequences with an average mass less than 25 were removed. Chimeric sequences were removed using USEARCH v7.1 (Edgar, 2010). Unique sequences with similarity of 97% or greater were clustered into operational taxonomic units (OTUs), using the UPARSE software (Edgar, 2013). The sequences were classified using the SILVA database containing bacterial and fungal ribosomal RNA sequences (version 119) (Pruesse et al., 2007). The Shannon index was calculated using mothur. High-throughput sequencing data have been deposited in the NCBI Sequence Read Archive (BioProject ID PRJNA360659, study accession number SRP097003).

### Statistical Analysis

One-way ANOVA of soil physical and chemical properties was performed using SPSS (version 19.0; SPSS, Chicago, IL, USA). Significance was calculated by Tukey’s test (*p* < 0.05). The Canoco program for Windows 4.5 (Biometris, Wageningen, the Netherlands) was used for principal component analysis (PCA). The relationship between soil microbial community structure and each affecting factor was analyzed by RDA and variation partitioning. RDA eliminates redundant variables depending on other measured variables, automatically selecting variables with large effects, and on the variance inflation factor values to gradually remove redundant parameters, and the significance levels are based on 999 Monte Carlo permutations. Linear discriminant analysis (LDA) coupled with effect size measurements (LEfSe) analysis was conducted to search for statistically different biomarkers between groups (Segata et al., 2011).

## Results

### Effects of Grazing Intensity on Soil Microbial Community Structure

DNA was extracted from soil samples of Loess Plateau grasslands with four different grazing intensities. The MiSeq platform was used for 16S/18S rRNA gene sequencing. A total of 662,030 and 623,204 quality-filtered and chimera-checked 16S/18S rRNA gene sequences were obtained with an average length of 438 and 402 bp across all samples, respectively. The number of 16S rRNA sequences obtained per sample varied from 10,079 to 24,997, and the number of fungal 18S rRNA sequences per sample varied from 10,267 to 29,908. In total, 2050 bacterial OTUs and 256 fungal OTUs were obtained from the 36 DNA samples.

**FIGURE 1:**
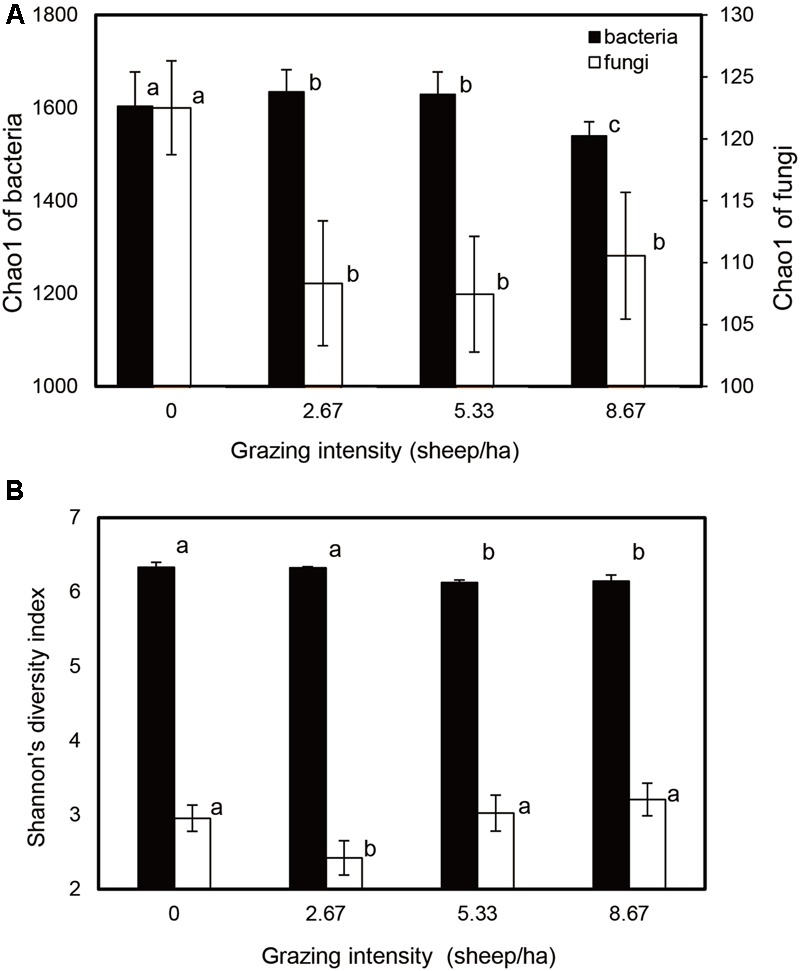
**Estimated values of bacterial and fungal community relative abundances (A)** and Shannon diversity index **(B)**. Significant differences are indicated by different letters.

Bacterial and fungal community relative abundances (Chao1) and diversity (Shannon; α-diversity) index values were compared for different grazing intensities (**Figure 1**). The Chao1 estimator indicated that bacterial community abundances in the S2.67 and S5.33 sample plots were significantly higher than those in S0 and S8.67; in addition, the community abundance in S0 was significantly higher than that in S8.67. The fungal community abundance was higher in the S0 sample plot than in the other plots (**Figure 1A**). The Shannon indices showed that the S0 and S2.67 bacterial communities were significantly more diverse than those of S5.33 and S8.67, while the fungal community was significantly less diverse in S2.67 than in the other three regimens.

**FIGURE 2:**
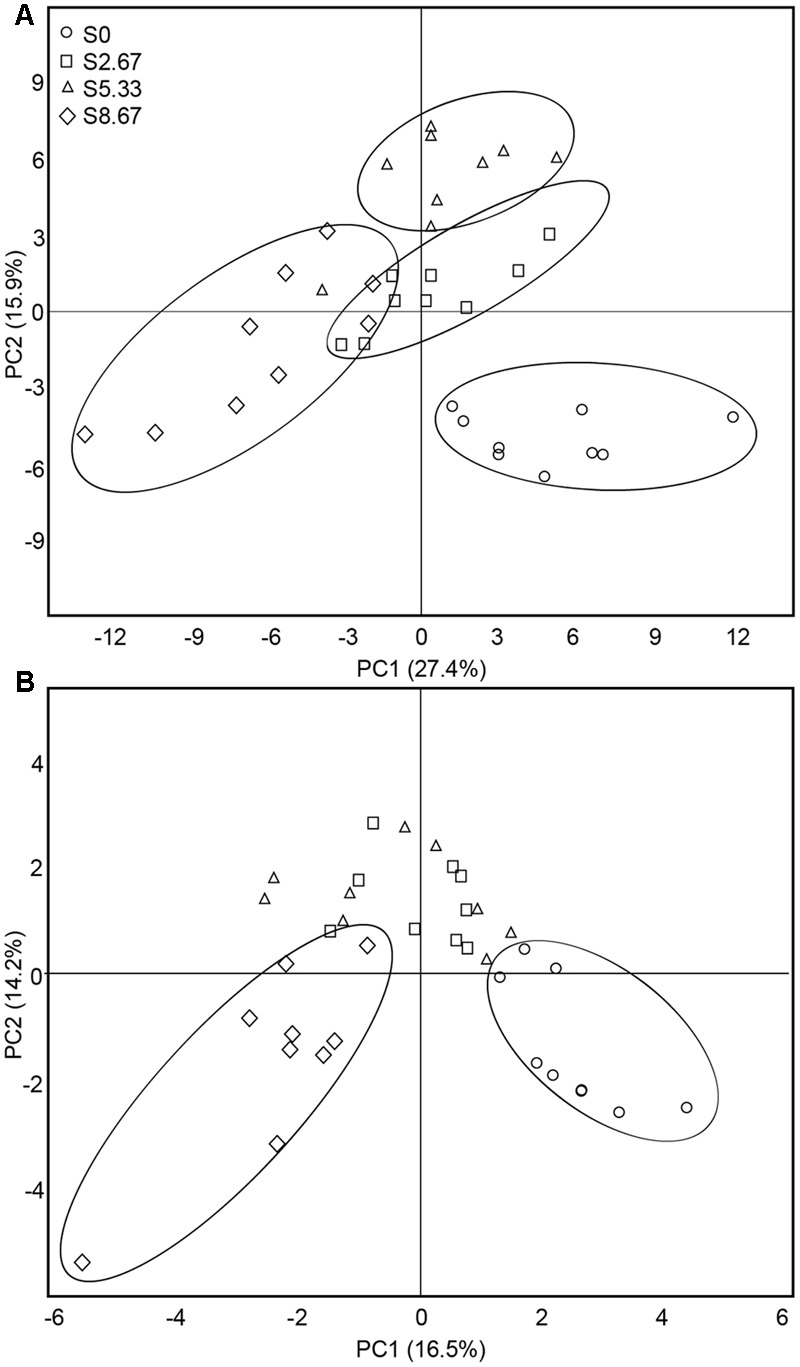
**Principal coordinates analysis of bacterial (A)** and fungal **(B)** communities. The values of axes 1 and 2 are the percentages that can be explained by the corresponding axis.

The predominant species in the bacterial and fungal communities were largely consistent among the four grazing regimens. However, differences in relative abundances were observed (Supplementary Figure S1). The bacterial phyla with high relative abundance were *Actinobacteria, Proteobacteria, Acidobacteria*, and *Chloroflexi*. The fungal phyla with high relative abundance were *Ascomycota* and *Basidiomycota*. The β-diversity was examined by PCA. The PCA clearly grouped the bacterial and fungal communities according to the four grazing regimens (**Figures 2A,B**). The first two axes (PC1 and PC2) explained 43.3 and 30.7%, respectively, of the total variance in the bacterial and fungal species in the four grasslands sampled.

### Relationship between Microbial Community Structure and Environmental Characteristics

The different grazing intensities had different effects on vegetation and soil characteristics (Supplementary Table S1). NH_4_-N was significantly lower in S8.67 than in the three other sample plots (*p* < 0.05), while the content of NO_3_-N was the highest in S0. Total nitrogen was significantly higher in S8.67 than in S2.67 and S5.33 (*p* < 0.05), but there was no significant difference with S0. The moisture content in S5.33 was significantly lower than that in S2.67 and S0 (*p* < 0.05), but it did not significantly differ from that in S8.67. Vegetation diversity in S8.67 was significantly higher than that in S2.67 and S0 (*p* < 0.05), whereas vegetation biomass in S8.67 was significantly lower than that in S2.67 and S0 (*p* < 0.05) (Supplementary Table S1).

**FIGURE 3:**
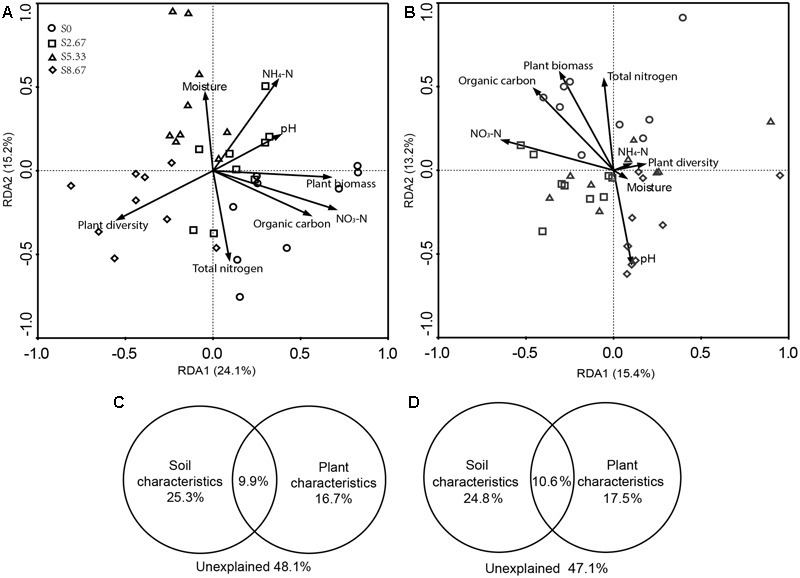
**Redundancy analysis (RDA) analysis of MiSeq data (symbols) and environmental characteristics (arrows).** Bacterial and fungal communities are shown in **(A,B)**, respectively. The values of axes 1 and 2 are the percentages explained by the corresponding axis. Analysis of the level of contribution of soil and plant characteristics to changes in bacterial **(C)** and fungal **(D)** communities.

Grazing changes microbial community structures and environmental characteristics. The disrupting effect of grazing on microbial communities may primarily be mediated by aboveground plant and soil geochemical characteristics. Therefore, this study investigated whether microbial community structure and environmental characteristics are related. RDA revealed that the microbial community structure was formed by primary environmental characteristics (including NH_4_-N, NO_3_-N, total nitrogen, organic carbon, moisture, pH, and vegetation biomass and diversity). After removal of the redundant variables, eight environmental characteristics were chosen for RDA. As shown in **Figures 3A,B**, plant biomass (*p* = 0.001), NO_3_-N (*p* = 0.04), and total nitrogen (*p* = 0.04) significantly affected the bacterial community structure, while NO_3_-N (*p* = 0.04) and total nitrogen (*p* = 0.04) significantly affected the fungal community structure.

Variance partition analysis was conducted to dissect the contributions of soil and plant characteristics to the microbial community structure. These selected characteristics together explained 51.9 and 52.9% of bacterial and fungal community changes, respectively (**Figures 3C,D**). The contribution of soil and plant characteristics explained 25.3 and 16.7%, respectively, of bacterial community changes and 24.8 and 17.5%, respectively, of the fungal community changes. In addition, the combined contribution of soil and plant characteristics explained 9.9 and 10.6% of the bacterial and fungal community changes, respectively, revealing a very close interaction between soil and plant characteristics.

### Microbial Communities with Statistically Significant Differences

Apart from determining α- and β-diversities, another primary goal of comparing microbial communities is to identify specialized communities in samples. To this end, we used the LEfSe tool (33). This tool allows analyzing microbial community data at any clade; however, as analysis of the large number of OTUs detected in this study would be computationally too complex, statistical analysis was performed only from the domain to the genus level.

**FIGURE 4:**
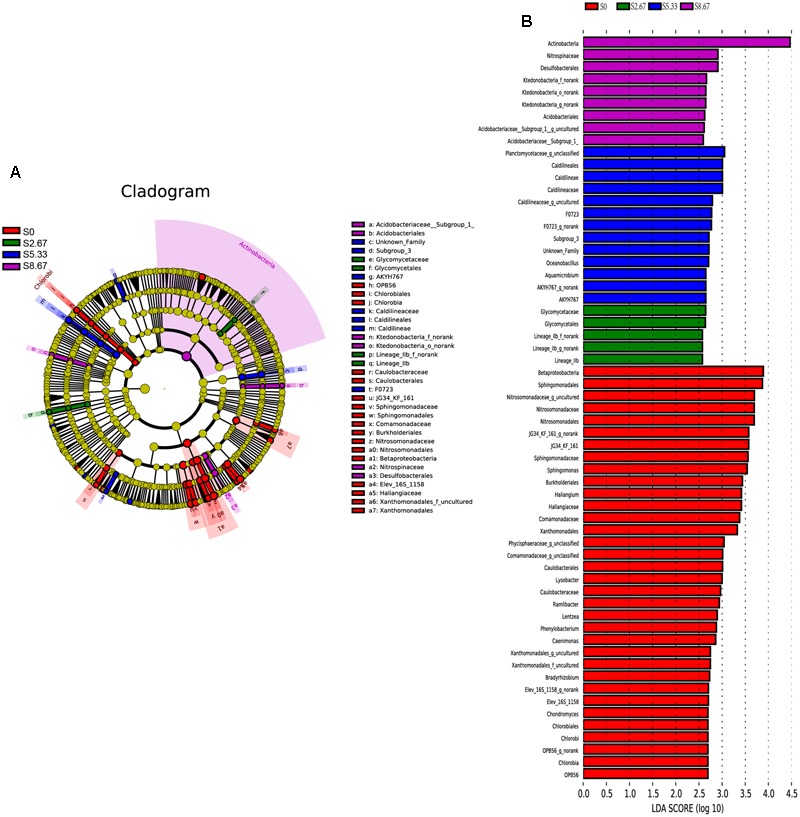
**Cladogram showing the phylogenetic distribution of the bacterial lineages associated with soil from the four grasslands with different grazing intensities (A)**. Indicator bacteria with LDA scores of 2 or greater in bacterial communities associated with soil from the four grasslands with different grazing intensities **(B)**. Different-colored regions represent different constituents (red, S0; green, S2.67; blue, S5.33; purple, S8.67). Circles indicate phylogenetic levels from domain to genus. The diameter of each circle is proportional to the abundance of the group.

**FIGURE 5:**
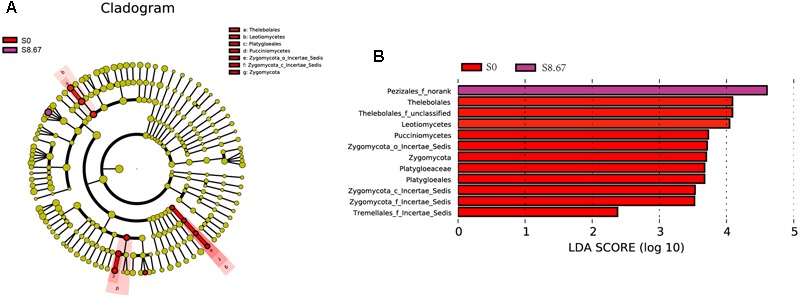
**Cladogram showing the phylogenetic distribution of the fungal lineages associated with soil from the four grasslands with different grazing intensities (A)**. Indicator fungi with LDA scores of 2 or greater in fungal communities associated with soil from the four grasslands with different grazing intensities **(B)**. Different-colored regions represent different constituents (red, S0; purple, S8.67). Circles indicate phylogenetic level from domain to family. The diameter of each circle is proportional to the abundance of the group.

Groups were shown in cladograms, and LDA scores of 2 or greater were confirmed by LEfSe (**Figures 4, 5**). In S0, six groups of bacteria and two groups of fungi were significantly enriched, namely *Chlorobi* (from phylum to genus), *Betaproteobacteria* (the class and orders of *Burkholderiales* and *Nitrosomonadales* to genus), *Caulobacterales* (from order to genus), *Sphingomonadales* (the order and families JG34_KF_161 and *Sphingomonadaceae*), *Xanthomonadales* (the order and its family), Elev_16S_1158, and *Haliangiaceae* (within *Deltaproteobacteria*), and *Pucciniomycetes* (the order and its family *Platygloeales*), and *Zygomycota* (from phylum to family) (**Figures 4, 5**). In S2.67, fewer microbes were significantly enriched; the bacteria *Glycomycetales* (the order and its family *Glycomycetaceae*) and Lineage_IIb (from order to genus; *Elusimicrobia*) were enriched, while no fungi were detected at a significant level (**Figures 4, 5**). In S5.33, four groups of bacteria and 1 group of fungi were detected to be significantly enriched, namely Subgroup_3 (the order and its family Unknown_Family), AKYH767 (order), *Caldilineae* (from class to genus), F0723 (order), and *Leotiomycetes* (from order to family) (**Figures 4, 5**). In S8.67, *Actinobacteria* was enriched only at the phylum level, and no significant enrichment was detected at any other clade. In addition, the enriched bacteria were *Acidobacteriales* (from order to genus), *Ktedonobacteria* (from order to genus), and *Desulfobacterales* (the order and its family *Nitrospinaceae*), and the only enriched fungi were *Pezizales* (*Pezizales*_f_norank), enriched at the family level (**Figures 4, 5**). Additionally, we used primers that detect other eukaryotic groups, such as *Chloroplastida, Amoebozoa, Holozoa, Alveolata, Discicristoidea*, and *Rhizaria*; however, no significant differences were found among the four grasslands with different grazing regimens (**Figure 5**).

## Discussion

In this study, four sample plots of grassland with different grazing intensities were set up in the Loess Plateau to analyze the relationships between soil bacterial and fungal communities and grazing intensity, and the interaction between plant communities and soil physical and chemical properties resulting from grazing intensity and bacterial and fungal communities. The predominant flora in the bacterial and fungal communities of Loess Plateau grassland soil was generally consistent between the four grazing regimens, but there were differences in relative abundance, and additionally, each regimen had its own unique microbial populations. Bacterial and fungal abundance and diversity were highest in S2.67 and S0, respectively. Similarly, Lienhard et al. (2013) found that maximum bacterial and fungal diversity occur with different utilization schemes. Microbial biomass and/or diversity can increase, decrease, or remain the same depending on the type of grassland, geographical location, and the grazing system and intensity (Ruess and McNaughton, 1987; Bardgett et al., 1997; Wang et al., 2006; Jangid et al., 2008; Zhou et al., 2010; Qi et al., 2011; Death and Barquín, 2012; Ford et al., 2013; Gou et al., 2015).

Although bacterial and fungal communities respond to grazing intensity in different ways with regard to relative abundances and diversity, our study showed that the soil microbial community structure changed significantly along a gradient of grazing intensity, which was consistent with changes in soil and plant characteristics. Although similar findings have been reported, these previous studies often relied on culture plate counting (Clegg, 2006; Wang and Sheng, 2012), phospholipid fatty acid analysis (Bardgett et al., 2001; Ingram et al., 2008), or denaturing gradient gel electrophoresis (Clegg, 2006) to determine the effects of grazing on microbes. With respect to microbe identification and classification, these methods may not be sufficiently accurate and/or are able to analyze only a limited set of microbial species in samples from complex environments and thus, do not allow a comprehensive understanding of the effects of grazing intensity on grassland microbial communities. As a consequence, inferences about regulators of the ecosystem and energy flow are limited when such methods are used (Chapin et al., 2000). Therefore, this study aimed to provide a more complete analysis of the microbial community in order to achieve a better understanding of the contributions made by the microbial communities.

We found that the environmental changes that occur with changes in grazing intensity contribute differently to different microbial groups in the community (**Figures 3A,B**). While shifts in bacterial communities showed a significant relationship with plant biomass, no such relationship was found in fungal communities. Zhou et al. (2010) found that livestock feeding affects aboveground vegetation biomass and community structure, and indirectly changes soil physical and chemical properties. This is a result of the interaction of microorganisms with plants (Ortiz-Castro et al., 2009). The fungal community structure in our study area was not as sensitive as the bacterial community structure to vegetation biomass changes. This may be because fungi are more likely to degrade lignocellulose from different plants than bacteria, allowing them to first obtain resources from many of the relevant available substances (Chapin et al., 2011). In addition, we found significant direct relationships of bacterial and fungal community structures with soil total nitrogen and NO_3_-N. With increasing grazing intensity, soil carbon and nitrogen storage decreases (Cui et al., 2005; He et al., 2008). In addition to soil total nitrogen and NO_3_-N, organic carbon also decreased, albeit not significantly, with the increase in grazing intensity in this study. Nitrogen is one of the most important nutrients for life; therefore, plant and microbial activities may gradually reduce the content of nitrogen in soil. However, correlations between microbial communities and environmental factors must be carefully explained because it is often very difficult to firmly establish the relationship between microbial communities and soil nutrient cycling (Bardgett, 2005). In addition, this study employed 16S rRNA and 18S rRNA sequencing to analyze microbial communities; the gene abundances indicate genetic potential and not necessarily microbial activity. Despite these limitations, which were considered in this study, we found that the environmental factors evaluated explained over 50% of the shift in the microbial communities, which suggests that they are the primary factors influencing microbial community structure. Multiple studies have shown that the effect of mild or moderate grazing on grassland soil is relatively small, and these grazing intensities are beneficial to dry matter production, nutrient cycling, and carbon and nitrogen storage (Han et al., 2008; Li et al., 2008; Chen et al., 2010). In this study, grazing in S2.67 had relatively little effect on grassland soil and plant characteristics, and with respect to bacterial communities, 2.67 sheep/ha can be considered a moderate stocking rate in the study region. According to the intermediate disturbance hypothesis (Connell, 1978), 2.67 sheep/ha may be close to the moderate-disturbance stocking rate in the Loess Plateau grassland.

Compared with the other regimens, ungrazed soil was the most enriched and showed the largest variety in indicator bacteria and fungi. One of the bacteria enriched in S0 was *Chlorobi*, also known as green sulfur bacteria, which are obligate anaerobic bacteria. However, some members of this family are facultative anaerobic and can degrade cellulose (Iino et al., 2010; Podosokorskaya et al., 2013). Presence of *Chlorobi* was significantly positively correlated with water-holding capacity and aboveground biomass; these parameters were significantly greater in S0 than in the other regimens (Supplementary Table S1). This could be owing to the air in the soil being saturated with water vapor, thus increasing anaerobicity, and/or plant litter, which increases the soil cellulose content. *Proteobacteria* was one of the most abundant phyla in all four grazing regimens, with the exception of *Desulfobacterales* enrichment in S8.67; the other significant clades were primarily enriched in S0. Most of the significantly enriched microbial clades in S0 showed a significant positive correlation with plant biomass (Supplementary Table S1). These microbial clades (*Betaproteobacteria, Caulobacterales, Sphingomonadales, Xanthomonadales, Leotiomycetes*, and *Zygomycota*) all have a close relationship with lignocellulosic degradation (Yoon et al., 2010; Štursová et al., 2012). Enrichment for *Caulobacterales* (Verastegui et al., 2014), *Sphingomonadales* (*Alphaproteobacteria*) (Abrusci et al., 2009), *Xanthomonadales* (*Gammaproteobacteria*) (Eichorst and Kuske, 2012), and *Zygomycota* (Chatzinotas et al., 2013) may be caused by a higher plant biomass in S0 than in the three other grasslands. In addition, *Sphingomonadales, Haliangiaceae*, and *Pucciniomycetes* also showed a significant positive correlation with OC, suggesting that increases in plant litter decomposition products or root secretions affect these microbial communities. Kindaichi et al. (2013) found that *Caldilineae* are effective phosphorus-removing bacteria that can absorb dissolved phosphorus from the environment and synthesize polyphosphates intracellularly. The soil nutrients in S5.33 may have created the most appropriate growth environment for *Caldilineae*, which might have accumulated the soil phosphorus in their cells, thereby significantly reducing the soil phosphorus content (Supplementary Table S1). S8.67 was primarily enriched for *Actinobacteria* and *Desulfobacterales*, and the fungal *Pezizales*_norank. No significant correlation was detected between *Actinobacteria* and the main environmental factors measured. This may be because correlations between higher taxonomic ranks and nutrient indicators may conceal relations with environmental parameters at the subfamily level (Cruz-Martínez et al., 2012). Total N was significantly increased in S8.67, which is easily explained by the fact that with increasing occupancy, the amount of excrement and consequently, soil nitrogen content, increases. A significant positive correlation was observed between *Desulfobacterales* and total N, which might be related to the nitrogen cycle; Widdel (1987) found that *Desulfobacter hydrogenophilus* fixates nitrogen, and *Nitrospinaceae* (also significantly enriched and a member of *Desulfobacterales*) show ammonia oxidation activity (Ionescu et al., 2012). The only significantly enriched fungi in S8.67 soil were *Pezizales*, which generally grow easily on disturbed soils (Petersen, 1985). Although some microbial taxa (such as *Tremellales_*incertae_sedis, Lineage_IIb, *Glycomycetales*, Subgroup_3, AKYH767, F0723, *Aquamicrobium, Oceanobacillus, Acidobacteriales, Ktedonobacteria_*norank) showed significant enrichment in different grazing intensities of the grassland, their contribution to their environment might be limited due to their low abundance (<0.1% of the sequences were from these taxa).

In summary, the response of soil microbial communities to grazing intensity was studied by partial 16S/18S rRNA gene analysis. MiSeq results showed that grazing changed the microbial community structure and increased bacterial community diversity and relative abundance in S2.67. Grazing intensity impacted the soil and vegetation characteristics to varying degrees, with relatively minimal impact in S2.67. In addition, changes in microbial communities were primarily attributable to plant biomass, and soil total nitrogen and NO_3_-N. Considering microbial community structure and environmental factors, a stocking rate of about 2.67 sheep/ha is appropriate for the semiarid Loess Plateau grassland evaluated in this study. Owing to the limited sampling and restricted sampling area, these conclusions cannot be generalized to other Loess Plateau grasslands; however, our findings represent an important step toward understanding how grazing has changed the soil microbial communities in the Loess Plateau grasslands. We are currently planning a more comprehensive study of the interaction between grazing activities and soil microbial communities in the Loess Plateau grassland system to formulate even more accurate recommendations for the conservation of the fragile grasslands of the Loess Plateau.

## Author Contributions

H performed the sampling, contributed to the experimental design, data analysis, and writing of the manuscript. XC contributed to the sampling and data analysis. FH provided to the study site. YW contributed to the data analysis and writing. YC contributed to the sampling, experimental design, data analysis, and writing of the manuscript.

## Conflict of Interest Statement

The authors declare that the research was conducted in the absence of any commercial or financial relationships that could be construed as a potential conflict of interest.
