# Supplementary material for: Bacterial and Fungal Community Structures in Loess Plateau Grasslands with Different Grazing Intensities
Source: Front Microbiol. 2017 Apr 7;8:606. doi: 10.3389/fmicb.2017.00606 (PMC5383705; doi:10.3389/fmicb.2017.00606)
Supplement: Supplementary file 1 [file Table_1.PDF]

Supplementary Material

Bacterial and Fungal Community Structures in Loess Plateau Grasslands with Different Grazing Intensities

Huhe, Xianjiang Chen, Fujiang Hou, Yanpei Wu, Yunxiang Cheng*

*** Correspondence:** Yunxiang Cheng: chengyx@lzu.edu.cn

Supplementary Table

Supplementary Table 1. ANOVA of soil from 4 different grazing regimens and plant characteristics and LEfSe analysis of correlation between significant biological groups. Total N (total nitrogen), OC (organic carbon), P-H (plant diversity), and P-B (plant biomass).

Sample plot or environmental factor	NH_4-N	NO_3-N	Total nitrogen	Organic carbon	Phosphorus	pH	Moisture	Plant diversity	Plant biomass
S0	0.89 ± 0.69a	5.78 ± 2.59a	0.62 ± 0.09b	5.1 ± 0.88a	0.42 ± 0ab	8.72 ± 0.03a	8.01 ± 0.51b	1.33 ± 0.08b	405.70 ± 55.32a
S2.67	0.89 ± 0.45a	4.2 ± 1.64ab	0.53 ± 0.12a	4.61 ± 1.17b	0.42 ± 0.04a	8.69 ± 0.08a	7.76 ± 1.01b	1.35 ± 0.37b	312.93 ± 66.89ac
S5.33	1.22 ± 0.53a	3.57 ± 1.04b	0.48 ± 0.06a	4.36 ± 0.7b	0.37 ± 0.04b	8.71 ± 0.02a	6.62 ± 0.98a	1.62 ± 0.18ab	284.17 ± 71.13bc
S8.67	0.61 ± 0.35b	3.93 ± 0.83b	0.6 ± 0.1b	4.38 ± 0.84b	0.39 ± 0.05ab	8.67 ± 0.05a	7.31 ± 1.26ab	1.89 ± 0.17a	199.93 ± 26.92b

Pearson correlation between environmental factors and significant groups (2-tailed)*

Significant group	Content(%)**	Correlation	Sig.	Correlation	Sig.	Correlation	Sig.	Correlation	Sig.	Correlation	Sig.	Correlation	Sig.	Correlation	Sig.	Correlation	Sig.	Correlation	Sig.
Chlorobi	0.06-0.1	-0.25	0.15	0.15	0.42	-0.04	0.81	0.19	0.28	-0.04	0.81	0.14	0.45	**0.55**	**0.00**	-0.22	0.21	**0.40**	**0.02**
Betaproteobacteria	2.6-4.2	0.33	0.06	0.15	0.41	-0.16	0.39	0.17	0.34	0.27	0.14	**0.39**	**0.03**	-0.04	0.81	**-0.72**	**0.00**	**0.55**	**0.00**
Caulobacterales	0.2-0.4	0.21	0.25	0.13	0.47	-0.09	0.64	0.11	0.55	**0.44**	**0.01**	0.31	0.07	-0.12	0.51	**-0.55**	**0.00**	0.32	0.06
Sphingomonadales	1.9-3.3	0.14	0.44	**0.44**	**0.01**	0.13	0.47	**0.35**	**0.05**	0.21	0.24	0.07	0.69	0.10	0.58	-0.31	0.07	**0.65**	**0.00**
Xanthomonadales	0.5-0.8	0.25	0.16	**0.38**	**0.03**	-0.09	0.62	0.23	0.21	0.02	0.92	0.27	0.12	0.03	0.88	**-0.45**	**0.01**	**0.66**	**0.00**
Elev-16S-1158	0.02-0.05	0.02	0.91	0.30	0.08	-0.20	0.27	-0.05	0.80	0.11	0.56	0.25	0.16	-0.09	0.62	**-0.60**	**0.00**	**0.32**	**0.06**
Haliangiaceae	0.07-1.2	0.30	0.09	**0.52**	**0.00**	0.22	0.22	**0.42**	**0.02**	**0.39**	**0.03**	-0.04	0.85	-0.06	0.73	**-0.47**	**0.01**	**0.63**	**0.00**
Lentzea	0.2-0.4	-0.09	0.64	-0.08	0.67	0.16	0.39	-0.04	0.83	0.27	0.12	0.15	0.42	0.02	0.91	-0.07	0.68	**0.38**	**0.03**
Zygomycota	1.1-2.3	0.01	0.98	-0.23	0.20	-0.25	0.16	-0.28	0.11	-0.30	0.09	0.34	0.06	-0.03	0.86	-0.11	0.55	**0.37**	**0.03**
Leotiomycetes	1.5-3.2	0.02	0.90	0.16	0.36	0.02	0.90	0.12	0.50	0.06	0.74	0.27	0.12	-0.06	0.76	**-0.41**	**0.02**	**0.51**	**0.00**
Pucciniomycetes	0-1.1	0.16	0.37	**0.57**	**0.00**	0.14	0.45	**0.36**	**0.04**	0.17	0.35	0.20	0.28	-0.01	0.96	**-0.49**	**0.00**	**0.45**	**0.01**
*Tremellales*_incertae_sedis	0-0.04	-0.15	0.40	0.03	0.89	0.02	0.90	-0.09	0.62	0.11	0.54	**-0.35**	**0.05**	0.12	0.49	0.10	0.59	-0.01	0.97
Lineage_IIb	0.01-0.04	-0.05	0.80	-0.04	0.85	-0.09	0.62	-0.09	0.63	0.10	0.58	0.15	0.40	0.02	0.93	-0.30	0.09	0.27	0.13
Glycomycetales	0-0.02	0.08	0.67	0.02	0.94	-0.19	0.28	-0.13	0.46	-0.15	0.42	0.14	0.45	-0.20	0.26	-0.10	0.60	0.07	0.70
Caldilineae	0.36-0.55	0.32	0.06	0.02	0.91	**-0.37**	**0.04**	-0.04	0.84	**-0.56**	**0.00**	0.17	0.33	**0.35**	**0.05**	-0.28	0.12	0.15	0.40
Subgroup_3	0.02-0.04	0.27	0.13	0.14	0.44	-0.34	0.06	0.19	0.30	-0.33	0.06	0.07	0.69	0.30	0.09	-0.11	0.53	0.11	0.55
AKYH767	0.01-0.03	0.31	0.08	-0.05	0.78	-0.25	0.16	0.07	0.70	-0.19	0.30	**0.37**	**0.04**	-0.10	0.57	-0.21	0.24	0.32	0.07
F0723	0-0.02	0.28	0.12	-0.15	0.41	**-0.43**	**0.01**	-0.23	0.20	-0.22	0.22	0.14	0.45	0.02	0.90	-0.02	0.91	-0.19	0.30
Aquamicrobium	0-0.04	0.13	0.46	-0.14	0.45	-0.33	0.06	-0.27	0.14	**-0.61**	**0.00**	0.23	0.19	**0.55**	**0.00**	0.18	0.33	0.02	0.93
Oceanobacillus	0-0.03	0.26	0.15	-0.22	0.23	**-0.39**	**0.03**	-0.13	0.46	**-0.51**	**0.00**	0.14	0.45	-0.17	0.35	0.04	0.81	-0.07	0.70
Actinobacteria	32-38	0.09	0.61	-0.33	0.06	-0.25	0.17	-0.23	0.21	-0.09	0.64	0.05	0.77	-0.26	0.14	-0.06	0.76	-0.04	0.84
Acidobacteriales	0-0.02	-0.30	0.09	0.16	0.37	0.26	0.15	-0.06	0.73	0.26	0.14	-0.32	0.08	-0.08	0.68	0.14	0.44	-0.16	0.38
*Ktedonobacteria*_norank	0-0.03	-0.32	0.06	-0.19	0.30	-0.05	0.80	-0.29	0.11	0.06	0.76	-0.10	0.60	0.01	0.95	0.23	0.19	**-0.34**	**0.05**
Desulfobacterales	0.2-0.4	0.83	0.49	-0.06	0.75	**0.33**	**0.05**	0.10	0.59	0.32	0.07	**-0.44**	**0.01**	-0.03	0.86	0.12	0.52	-0.10	0.58
*Pezizales*_norank	1.2-8.8	0.39	0.83	-0.33	0.06	-0.15	0.40	-0.19	0.28	-0.27	0.13	0.09	0.64	0.28	0.12	0.21	0.23	-0.18	0.31

*** Boldface is used for a significance of < 0.05.**

**** Content of significant microbial groups in each clade.**
