# Supplementary material for: Bacterial and Fungal Community Structures in Loess Plateau Grasslands with Different Grazing Intensities
Source: Front Microbiol. 2017 Apr 7;8:606. doi: 10.3389/fmicb.2017.00606 (PMC5383705; doi:10.3389/fmicb.2017.00606)

Supplementary Material

Bacterial and Fungal Community Structures in Loess Plateau Grasslands with Different Grazing Intensities

Huhe, Xianjiang Chen, Fujiang Hou, Yanpei Wu, Yunxiang Cheng*

*** Correspondence:** Yunxiang Cheng: chengyx@lzu.edu.cn

Supplementary Figure

Supplementary Figure 1. Double hierarchical clustering analysis heat map of bacterial (A) and fungal (B) phyla in grasslands with 4 different grazing intensities. The neighbor-joining method was adopted to calculate bacterial and fungal phylogenetic trees, and the relationships between samples were confirmed using the Bray–Curtis distance and the complete clustering method. Each block in the heat map represents the relative abundance (%) of each bacterial and fungal phylum. The color scale at the bottom of the figure indicates the relative content of bacteria and fungi.

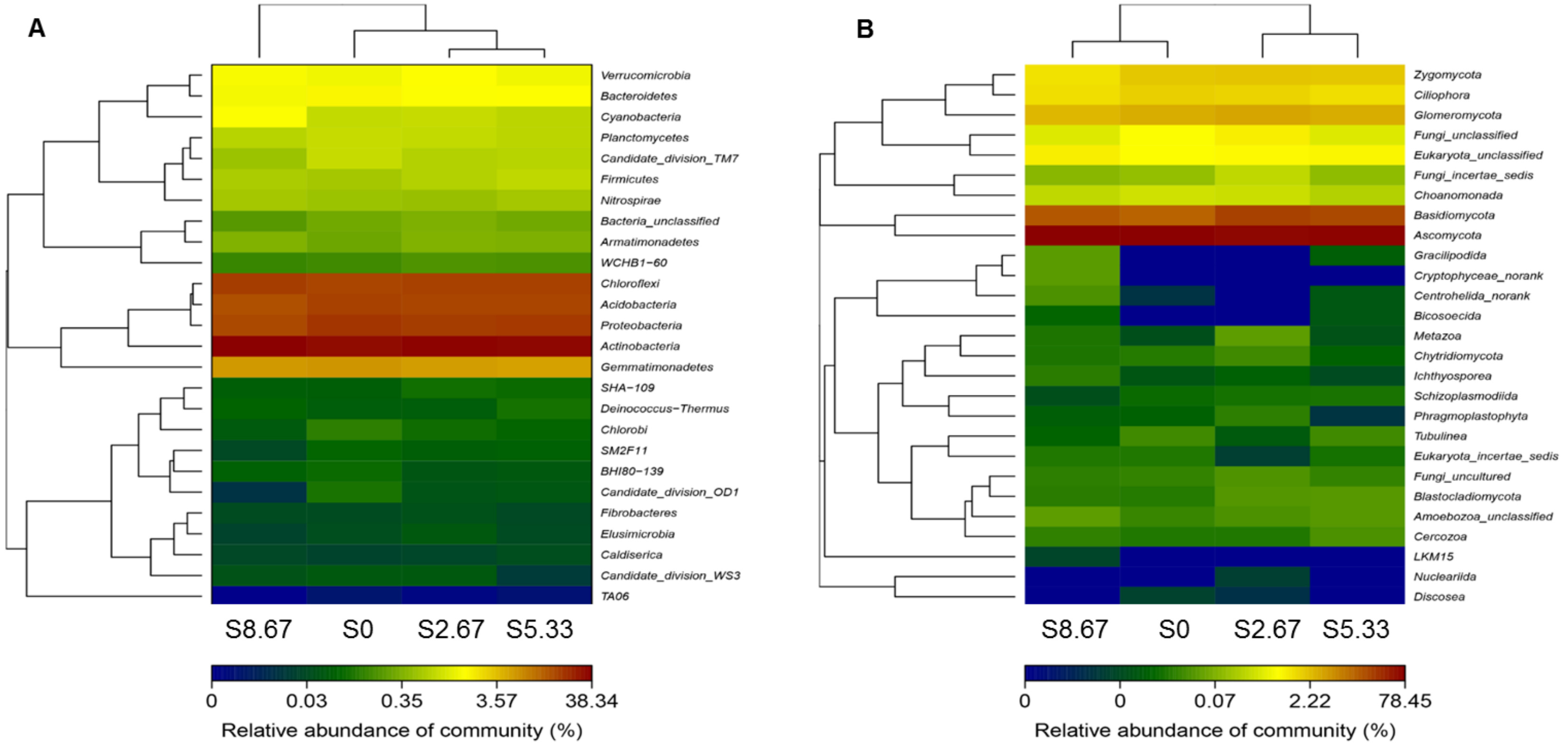

Supplement: Supplementary file 2 [file Image_1.PDF]
